# Supplementary material for: The Role of Ultrasound in the Evaluation of Inguinal Lymph Nodes in Patients with Vulvar Cancer: A Systematic Review and Meta-Analysis
Source: Cancers (Basel). 2022 Jun 23;14(13):3082. doi: 10.3390/cancers14133082 (PMC9265034; doi:10.3390/cancers14133082)

Supplementary Material Data S1

Search Strategy

Pubmed

((("Vulvar Neoplasms"[Mesh] OR "vulvar cancer" OR "vulvar cancers" OR "vulva cancer" OR "vulva neoplasm" OR "Vulvar carcinoma" OR "vulvar malignancy" OR "vulvar malignancies" OR "vulvar squamous cell Carcinoma" OR "vulvar melanoma" OR "vulvar adenocarcinoma" OR "vulvar basal cell carcinoma") OR ("groin recurrence" OR "groin metastasis" OR "inguinal node metastasis" OR "Inguinal lymph node metastases" OR "inguinal lymph node" OR "inguinal sentinel lymph node" OR " inguinal sentinel node" OR "inguinal sentinel node biopsy" OR "inguinal lymph node biopsy" OR " inguinofemoral lymphadenectomy")) AND ("Ultrasonography"[Mesh] OR ultrasound OR "Ultrasonic Tomography" OR "Computer Echotomography" OR "Ultrasonic Diagnosis" OR Echography OR "Ultrasonographic Imaging" OR "Ultrasound Imaging" OR "Diagnostic Ultrasound" OR Echotomography OR Sonography))

Filter only humans, only english

Web of Science

TS=((("Vulvar Neoplasms" OR "vulvar cancer" OR "vulvar cancers" OR "vulva cancer" OR "vulva neoplasm" OR "Vulvar carcinoma" OR "vulvar malignancy" OR "vulvar malignancies " OR "vulvar squamous cell Carcinoma" OR "vulvar melanoma" OR "vulvar adenocarcinom a" OR "vulvar basal cell carcinoma") OR ("groin recurrence" OR "groin metastasis" OR "inguinal node metastasis" OR "Inguinal lymph node metastases" OR "inguinal lymph node" OR "inguinal sentinel lymph node" OR " inguinal sentinel node" OR "inguinal sentinel node biopsy" OR "inguinal lymph node biopsy" OR " inguinofemoral lymphadenectomy")) AND ("Ultrasonography" OR ultrasound OR "Ultrasonic Tomography" OR "Computer Echotomography" OR "Ultrasonic Diagnosis" OR Echography OR "Ultrasonographic Imaging" OR "Ultrasound Imaging" OR "Diagnostic Ultrasound" OR Echotomography OR Sonography))

Indexes=SCI-EXPANDED, SSCI, A&HCI, CPCI-S, CPCI-SSH, ESCI Timespan=All years

No filters

SCOPUS

(((*"Vulvar Neoplasms" [mesh]* OR *"vulvar cancer"* OR *"vulvar cancers"* OR *"vulva cancer"* OR *"vulva neoplasm"* OR *"Vulvar carcinoma"* OR *"vulvar malignancy"* OR *"vulvar malignancies"* OR *"vulvar squamous cell Carcinoma"* OR *"vulvar melanoma"* OR *"vulvar adenocarcinoma"* OR *"vulvar basal cell carcinoma"*) OR (*"groin recurrence"* OR *"groin metastasis"* OR *"inguinal node metastasis"* OR *"Inguinal lymph node metastases"* OR *"inguinal lymph node"* OR *"inguinal sentinel lymph node"* OR *" inguinal sentinel node"* OR *"inguinal sentinel node biopsy"* OR *"inguinal lymph node biopsy"* OR *" inguinofemoral lymphadenectomy"*)) AND (*"Ultrasonography" [mesh]* OR *ultrasound* OR *"Ultrasonic Tomography"* OR *"Computer Echotomography"* OR *"Ultrasonic Diagnosis"* OR *echography* OR *"Ultrasonographic Imaging"* OR *"Ultrasound*

Imaging" OR *"Diagnostic Ultrasound"* OR *echotomography* OR *sonography*)) AND (LIMIT-TO (LANGUAGE , *"English"*))

Filter: only English

Supplementary Material Figure S1: Funnel plot evaluating publication bias

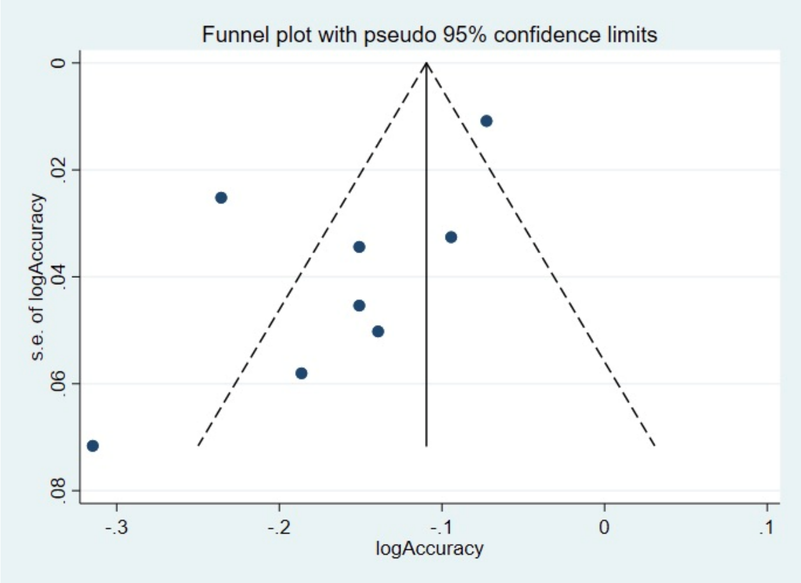

Supplement: Supplementary file 1 [file cancers-14-03082-s001.zip › cancers-1758838-supplementary.pdf]
